# Pilot feasibility study of an emergency paediatric kit for intra-rectal quinine administration used by the personnel of community-based health care units in Senegal

**DOI:** 10.1186/1475-2875-6-152

**Published:** 2007-11-15

**Authors:** Jean Louis A Ndiaye, Roger C Tine, Babacar Faye, El Hadj Lamine Dieye, Pape Amadou Diack, Valérie Lameyre, Oumar Gaye, Husseyn Dembel Sow

**Affiliations:** 1 Service de Parasitologie, Faculté de Médecine, Université Cheikh Anta DIOP de Dakar, Senegal; 2 Hôpital d'Enfants Albert Royer, Center Hospitalier National Universitaire de Dakar Fann, Senegal; 3 Ministère de la Santé et de la Prévention Médicale, Senegal; 4 Impact Malaria, Sanofi-Aventis Group, Gentilly, France

## Abstract

**Background:**

Quinine injection is the reference treatment for malaria when oral administration is impossible. Quinine can also be administered by the intra-rectal route and, over the last ten years, a series of studies have been conducted in children to determine the ideal dose and dilution in the African situation. The aim of the present study was to evaluate the feasibility and usefulness of a kit for an immediate administration of quinine alkaloids (Quinimax^®^) by community health workers, prior to transfer of the child to a more sophisticated health care establishment.

**Methods:**

A prospective, open, descriptive community intervention study conducted in northern Senegal at six village Health Units in children fewer than ten years of age with non-per-os malaria. Controls were given the routine care prior to transfer to a Health Center, and cases were in addition administered Quinimax^® ^(20 mg/ml) via the intra-rectal route before transfer. Patients were followed through complete cure and parasitological tests were carried out on Days 0, 3 and 7.

**Results:**

134 patients (79 cases/55 controls) were recruited between November 2003 and May 2004 or October and November 2004. The two groups were comparable at inclusion. In the case group, oral drugs could be administered after a mean of *16.8 hours *versus *33.6 hours *in the control group. Time-to cure was shorter in cases than in controls. Complete parasite clearance was obtained in all patients by Day 7. The kit was well accepted by all concerned and more than 80% of community health workers judged the kit easy to use.

**Conclusion:**

The emergency paediatric kit is a useful tool in the management of malaria in children who cannot be treated orally. It is feasible and easy to use for health workers in community-based Health Units where, according to the WHO, nearly 80% of malarial morbidity and mortality occurs.

## Background

Malaria is the most serious parasitic disease throughout the tropics. According to the World Health Organization (WHO), more than two billion people are exposed, most of them living in Africa, the Americas and Southeast Asia. Malaria accounts for about 25% of all children's deaths in Africa [1]. Every year, more than 300 million people contract acute malaria and, of these new clinical cases, 1.7–2.4 million die (mostly children). In Africa, nearly 80% of children undergoing a severe malaria attack die before they reach any health care establishment, and only a minority is given any specific treatment [2].

The recommended treatment for severe malaria includes the injection, ideally intravenous, of quinine salts as soon as the first signs of severe malaria or danger manifest (WHO). However, community health structures possess neither the equipment nor the expertise to perform this type of procedure. The situation has to be improved so that all children who develop severe malaria can be treated as quickly as possible, beginning close to home. Intra-rectal administration could provide a solution to these problems. When oral administration is impossible, community-based workers in remote village health care units could administer treatment via this route, prior to transferring the patient to a more sophisticated establishment for comprehensive management.

A number of studies conducted in various countries in Africa (Niger, Burkina Faso, Madagascar and Senegal) have shown that intra-rectal administration of diluted quinine is an effective, safe form of treatment in the management of malaria in children [3-6].

In Senegal, the health care system in more remote areas is based on village-based health units run by community health workers with no formal medical or paramedical qualifications.

The aims of this study were (i) to study the feasibility of an emergency pediatric kit when used by community health workers; (ii)to evaluate the acceptance of this form of treatment; (iii) to evaluate the therapeutic benefit of immediate intra-rectal quinine administration in children with malaria who cannot be treated orally (*non per os *malaria).

## Methods

### Study site

The village of Savoigne is located thirty kilometers north-east of Saint Louis in the Senegal River Delta (Richard-Toll district). The climate is Sahelian with a long dry season between November and June, and a short rainy season from July to October.

Malaria is unstable in this region with great variability from one spot to another. The main vectors are *Anopheles pharoensis *and *Anopheles gambiae *s.l. [7,8]. The Plasmodium Index ranges from 5.20% to 38.50% in under-15 year-olds [9]. According to clinical data collected in the Richard-Toll district in 2003, it is estimated that malaria accounts for 35% of all local pathology.

### Study design

A prospective, descriptive community intervention study was conducted in children consulting community health workers in six villages around the Savoigne Health Center (Table 1). Most of the workers running the village-based units have only primary school education, and they are helped by local, unqualified midwives.

**Table 1:** Health Units around the Savoigne Health Center

Health Unit	Distance from Health Center	Population
Ndelle*	4 km	500 inhabitants
Barry	6 km	425 inhabitants
Ndiaye	7 km	1011 inhabitants
Ndioungue*	8 km	848 inhabitants
Mbodiène	9.5 km	912 inhabitants
Diagambal*	12.5 km	1086 inhabitants

* cases villages where intra rectal quinine was given

The six villages were randomized into two groups of three. The first group named a control group in which the children suffering from severe or *non per os *malaria were given conventional treatment before referral to the Health Center contains 3 villages. The second group of 3 villages named case group (or intervention group) in which the children received intra-rectal quinine before transfer. These 2 groups were made in order to describe the reference towards the health center whether or not a administration of quinine alkaloids was done to children with a *non per os *or severe malaria attacks in a same area at the same time. In order to investigate whether or not the supplementary treatment delayed referral, the research team did not get involved with the transfer of the sick children to the Health Center.

Included were all children of under 25 kilograms and/or 10 years of age presenting with a suspected malarial attack who could not be orally treated (severe or *non per os *malaria). Children in whom intra-rectal quinine administration was contra-indicated (because of diarrhoea or pre-existing anal disease) were excluded from the case Group. Parental consent was obtained before enrolment.

After treatment at the village-based unit, all parents were told to take their sick children to the Health Center for confirmation of the diagnosis, curative treatment and follow-up.

This Study was approved by the Senegalese Ethics & Scientific Committee on October 22, 2003.

### Procedures

In the control villages, the children were cared for in line with national guidelines (paracetamol for high fever if possible and referral). In the case villages, the included children were also given an intra-rectal dose of 20 milligrams of quinine alkaloids per kilogram body weight (diluted in water to 30 mg/ml). The paediatric kit contains;

• one phial of 4 ml wich contains 500 mg of quinine alkaloids composed by : 480 mg of quinine (770,25 mg of quinine gluconate), 13,2 mg of quinidine (21,18 mg of quinidine gluconate), 3,4 mg of cinchonine (4,24 mg of cinchonine chlorhydrate) and 3,24 mg of cinchonidine (4,03 mg of cinchonidine chlorhydrate). This quinine is to be diluted into

• A bottle of 13,5 ml of purified water,

• a syringe graduated according to children bodyweight and age wich to administrate by rectal route the reconstituted solution by rectal route

The child was then monitored for thirty minutes. During this period, if any of the product was discharged, a further half-dose was administered before referral.

On arrival at the Health Center, a blood smear was performed to confirm the diagnosis and, for those with demonstrated malaria, treatment was administered in accordance with current National Malaria Control Programme recommendations (IV quinine or referral to the Regional Hospital). As soon as possible, the switch was made to an oral regimen with the currently recommended combination (SP + amodiaquine). At the Health Center, the patient's clinical condition was monitored and parasitological tests were performed every day until oral drugs could be administered. The patients were followed up through at least Day 7.

### Statistical analysis

Two populations were analysed: Population 1 covering all eligible patients included at the village units who were referred to the Health Center; and Population 2 covering all eligible patients included at the village units who were found to have a confirmed parasitaemia on arrival at the Health Center.

The statistical analysis was performed using SAS software Version 8.2 (SAS Institute – Cary, NC). Descriptive analyses were carried out for each treatment group. The statistical significance threshold was set at 5%. The analysis describe the time-to referral and clinical outcome in the intervention zone (case group with intra rectal quinine) versus the control group or non intervention zone.

## Results

A total of 134 children (79 in case villages and 55 in control villages) were included in two phases, the first between November 2003 and May 2004, and then from October to November 2004.

### Description of the two groups at inclusion

At inclusion, demographic characteristics were the same in the two groups (Table 2)

**Table 2:** Population details

	Cases N = 79	Controls N = 55	Total N = 134
**Gender**			
Male	43 (54.4%)	30 (54.5%)	73 (54.5%)
Female	36 (45.6%)	25 (45.5%)	61 (45.5%)

**Ethnic origin**			
Peulh	9 (11.4%)	8 (14.5%)	17 (12.7%)
Woolof	69 (87.3%)	46 (83.6%)	115 (85.8%)
Serere	1 (1.3%)	0	1 (0.7%)
Diola	0	1 (1.8%)	1 (0.7%)

**Age (years)**			
Mean (SD)	4.6 (2.6)	4.7 (2.7)	4.7 (2.6)
Median [Q1; Q3]	4.0 [2.5;6.0]	4.5 [2.7;6.0]	4.0 [2.5;6.0]
(Minimum; Maximum)	1;10	1;10	1;10

**Weight (Kg)**			
Mean (SD)	13.85 (4.41) 13.00	13.18 (4.67) 12.00	13.58 (4.52) 13.00
Median [Q1; Q3]	[10.20;17.00]	[10.00;16.00]	[10.00;16.00]
(Minimum; Maximum)	7.5;25.3	4.0;27.0	4.0;27.0

**Temperature (°C)**			
Mean (SD)	38.82 (1.08) 39.20	38.55 (1.49) 38.90	38.71 (1.27) 39.05
Median [Q1; Q3]	[38.40;39.40]	[38.10;39.50]	[38.40;39.50]
(Minimum; Maximum)	34.9;40.5	32.7;40.2	32.7;40.5

The time elapsed since appearance of the first symptoms was the same (mean = 52 hours in both groups) and symptoms were similar – exceptions were the incidences of: hallucinations reported by 32.9% (n = 26) cases versus 45.5% (n = 25) controls; and agitation reported by 51.9% (n = 41) cases and 70.9% (n = 39) controls (Table 3).

**Table 3:** Symptoms at inclusion

Symptom	Cases N = 79	Controls N = 55	Total N = 134
**Vomiting**			
Yes	66 (83.5%)	39 (70.9%)	105 (78.4%)
Non	13 (16.5%)	16 (29.1%)	29 (21.6%)

**Convulsions**			
Yes	3 (3.8%)	7 (12.7%)	10 (7.5%)
Non	76 (96.2%)	48 (87.3%)	124 (92.5%)

**Loss of consciousness**			
Yes	2 (2.5%)	4 (7.3%)	6 (4.5%)
Non	77 (97.5%)	51 (92.7%)	128 (95.5%)

**Agitation**			
Yes	41 (51.9%)	39 (70.9%)	80 (59.7%)
No	38 (48.1%)	16 (29.1%)	54 (40.3%)

**Hallucinations**			
Yes	26 (32.9%)	25 (45.5%)	51 (38.1%)
No	53 (67.1%)	30 (54.5%)	83 (61.9%)

**Bloody feces**			
Yes	4 (5.1%)	2 (3.6%)	6 (4.5%)
No	75 (94.9%)	53 (96.4%)	128 (95.5%)

**Anal lesion**			
Yes	0	2 (3.6%)	2 (1.5%)
No	79 (100.0%)	53 (96.4%)	132 (98.5%)

### Feasibility and acceptance of the kit (79 Cases)

After pre-calibrated dilution of the Quinimax^® ^solution, the health worker withdrew the volume corresponding to the child's weight (using a syringe with kilogram graduations).

The administered dose was considered correct if the dose administered corresponded exactly to the child's weight: it was found to be correct in 93.6% of cases (and the deviations detected in the remaining cases were small)(Table 4).

**Table 4:** Dosing and ease of administration

	Village cases
**Correct dose**	Yes (93.6%)
	No (6.4%)

**Ease of administration**	Easy (77.2%)
	Difficult (22.8%)

**Quantity administered (kg eq.)**	
Mean (SD)	13.87 (4.43)
Median [Q1; Q3]	13.00 [10.00;17.00]
(Minimum; Maximum)	7.5;25.0

Administering the product was deemed easy in 77.2% of cases, and some of the difficulty of administration may be related the patients' reactions which were observed in 39% (n = 31), including agitation, crying out and tears (probably due to fear on introduction of the device). No parental reactions were reported. In 25% of cases, early discharge of the product within 30 minutes of the first dose necessitated the administration of a further half-dose of intra-rectal Quinimax^®^.

### Time-to transfer from the Health Unit to the Health Center (n = 134 children)

All 134 patients were referred to the Savoigne Health Center. The mean time-to transfer in this rural area was 4.40 hours (± 11.07) in the case group, and 3.11 hours (± 5.76) in the control group for the parents to bring children from their villahe to the health post.

### Therapeutic benefit

This end point was evaluated by comparing the cases and the controls with a confirmed parasitaemia (n = 92) vis-à-vis fever and parasitaemia over time, as well as the time intervals to obtain cure, to stop vomiting and to the switch to oral drugs.

### Time-to cure

**Figure 1:**
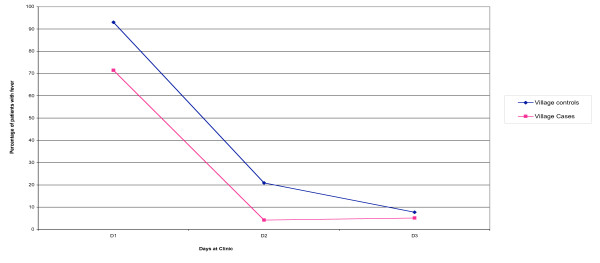
Percentage of patients with fever.

On Day 1, fewer cases than controls had fever (71.4% versus 93.0%). This difference persisted through Day 2 but had disappeared by Day 3 (Figure 1).

Vomiting stopped within one day in 97.8% cases and 79.5% controls (Table 5).

**Table 5:** Time-to stoppage of vomiting

Number of days to the stoppage of vomiting			
All included patients	Cases	Controls	Total N = 134

≤1 day	71 (95.9%)	38 (76.0%)	109 (87.9%)
> 1 day	3 (4.1%)	12 (24.0%)	15 (12.1%)

patients with a confirmed parasitemia	Cases N = 49	Controls N = 43	Total N = 92

≤1 day	45 (97.8%)	31 (79.5%)	76 (89.4%)
> 1 day	1 (2.2%)	8 (20.5%)	9 (10.6%)
Unknown	3	4	7

The mean number of days between the day of the consultation (and intra-rectal quinine administration at the village Health Unit for the cases) and the switch to oral drugs was significantly shorter in the case group than in the control group (0.7 days versus 1.4 days) (Table 6).

**Table 6:** Time-to switch to oral drugs

**Time-to switch (days)**	Cases	Controls	Total
N	47	42	89
Mean (SD)	0.7 (0.8)	1.4 (0.7)	1.0 (0.8)
Median [Q1;Q3]	1.0 [0.0;1.0]	1.0 [1.0;2.0]	1.0 [1.0;1.0]
Minimum;Maximum	0;5	1;4	0;5

Similarly, the interval between consultation and cure was shorter in the case group than in the control group (1.9 days versus 2.2 days;).

### Parasite load

**Figure 2:**
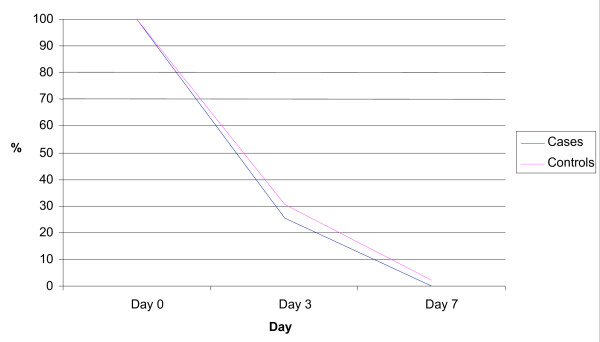
Percentage of patients with parasitemia from Day 0 to Day 7.

On Day 3, blood smear were carried out in the 92 patients with parasitaemia on arrival at the Health Center. Parasite clearance was recorded in 74.4% of cases and 69.2% of controls (NS) (Figure 2).

Clearance was obtained in all patients by the end of the follow-up period.

### Safety of intrarectal Quinimax

Safety data (symptoms) collected at the Health Center were analysed for all included patients.

On arrival at the Health Center, a difference was observed between the two groups in the mean number of feces in the preceding 24 hours: 1.2 in the case group versus 0.1 in the control group (p < 0.0001).

Of the village children who passed a stool on arrival at the Health Center (78 including six controls), eight cases passed liquid stools compared with three controls (Table 7).

**Table 7:** Number and nature of feces at inclusion and on arrival at the Health Center

	Cases	Controls
**Number of feces in the preceding 24 h**		
Mean (SD)	0.6 (0.9)	1.0 (1.0)
Median [Q1; Q3]	0.0 [0.0;1.0]	1.0 [0.0;2.0]
(Minimum; Maximum)	0;4	0;4

**Number of feces on arrival at the Health Center**		
Mean (SD)	1.2 (0.9)	0.1 (0.4)
Median [Q1; Q3]	1.0 [1.0;2.0]	0.0 [0.0;0.0]
(Minimum; Maximum)	0;5	0;2

**Nature of the feces on arrival at the Health Center**		
Diarrhea: thin or watery feces (3 or +)	1 (1.6%)	0
Thin or watery feces (fewer than 3)	7 (11.3%)	3 (50.0%)
Soft, pale feces	47 (75.8%)	3 (50.0%)
Normal stools	7 (11.3%)	0

In the following days (Day 2 through Day 5), the number of faeces per day in the two groups became comparable (Table 8).

**Table 8:** Bloody feces before and after treatment

Day	Cases	Controls
Day 0: arrival at the Health Unit	**4**	**2**
Day 1: arrival at the Health Center	**2**	**2**
Day 2 to Day 5	6	3

### Serious Adverse Events

In the course of this Study, one control child died after having been referred from the Health Center to Saint Louis Regional Hospital (the referral center for severe cases) with severe dehydration and clinical anaemia.

## Discussion

This study was conducted to evaluate the feasibility and safety of an emergency paediatric kit when used by unqualified, community-based personnel, and to assess acceptance of the kit by local people.

The community-based health workers were observed to appreciate this novel therapeutic device.

Malaria was incorrectly diagnosed by community health workers in 22% of cases in this Study. The mean percentage of mistaken diagnoses has been estimated at 45% in certain health care structures in Senegal [10], and at 62% at the Muraz Center (Bobo Dioulasso/Burkina Faso) in the context of severe malaria [11]. This suggests that regular training and close supervision can improve the accuracy of malaria diagnosis by community health workers.

The administration of treatment prior to referral did not delay transfer of children to a more sophisticated health care facility for parenteral management.

The emergency paediatric kit is an effective tool in the early management of malaria in children who cannot be treated orally. Its use significantly cut down the period of time before the switch could be made to oral drugs, and accelerated clinical cure.

The percentage of children in whom early discharge of the administered product was observed was higher in this study (25%) than those reported by Barennes (12.9%)[2] and Ndiaye (12.1 %) [12]. Such discharge within thirty minutes will cut down the concentration of quinine in the blood but this can be compensated by administering another half-dose [13]. No particular adverse reactions were noted in patients who were given the second administration. Compression of the buttocks for five minutes after administration reduces the risk of discharge.

No serious adverse reaction to the product was observed in the course of this study, and none had been reported in either Madagascar, where intrarectal quinine administration has been common since 1985 [14] or in clinical trials in Niger, Togo, the Congo and Senegal [15,12]. In Niger, where a policy based on intrarectal quinine administration was officially adopted in 1994, Harouna reported a major anal abscess following this form of treatment in a patient who had previously experienced severe complications following intramuscular quinine injection; the abscess was cured with treatment [16].

## Conclusion

The paediatric kit used in this study provides a useful therapeutic option for early, community-based malaria management in children who cannot be treated orally, until they can be transferred to a more sophisticated health care establishment for definitive care. It is feasible at the community level and simple to use. Its generalization should be accompanied by the training of community health workers on how to use it, as well as its specific indications and contraindications.

## Competing interests

The author(s) declare that they have no competing interests.

## Authors' contributions

JLAN: designed the study, collected data, and prepared the manuscript

RCT: collected data, and prepared the manuscript

BF : contributed in the preparation of the manuscript

VL: designed the study and prepared the manuscript

ELD and PAD: collected data and participated in the design of the study

OG: designed the study and prepared the manuscript

HDS: designed the study and prepared the manuscript

All authors read and approved the final manuscript
